# The Impact of Hospital Competition on the Quality of Care in Europe: A Systematic Review

**DOI:** 10.3390/healthcare12222218

**Published:** 2024-11-06

**Authors:** Yuriy Timofeyev, Viktoriya Goldenova, Elza Mantaeva, Mihajlo Jakovljevic

**Affiliations:** 1 Graduate School of Business, HSE University, 119049 Moscow, Russia; 2 Department of State and Municipal Management and Law, Kalmyk State University named after B.B. Gorodovikov, 358000 Elista, Russia; 3 UNESCO-TWAS, The World Academy of Sciences, 34151 Trieste, Italy; 4 Shaanxi University of Technology, Hanzhong 723001, China; 5 Department of Global Health Economics and Policy, University of Kragujevac, 34000 Kragujevac, Serbia

**Keywords:** competitiveness, market pressure, rivalry, hospital care, care quality, Europe

## Abstract

**Objectives**: This study analyzes the results of empirical studies on the impact of hospital competition (rivalry and market pressure) on the quality of care in European countries. **Methods**: A systematic review has been conducted according to the Preferred Reporting Items for Systematic Reviewing and Meta-Analysis (PRISMA) guidelines, using the following online databases: PubMed, ScienceDirect, Wiley Online Library, and Google Scholar. The search protocol covers studies published in English between January 2015 and mid-April 2024. **Results**: Eight out of 14 eligible studies document significant positive associations, at least in the short term, between hospital competition and the quality of care measured through objective outcome indicators. Of the other six, one study demonstrates a negative relationship in a specific context. The findings of the remaining five studies are heterogeneous and context-dependent (two out of five) or suggest no discernible association between the two examined phenomena (three out of five). The respective contexts with positive, negative, or no statistically significant associations have been identified. **Conclusions**: The most essential impacts of competition on the quality of hospital care have been summarized, and avenues for future research and policy implications have been discussed.

## 1. Introduction

In accordance with economic theory, competition effectively mobilizes production [1]. Competitive markets create the necessity for manufacturers to constantly improve technologies and processes in a relentless pursuit to lower costs. New technologies catalyze competition by diffusing widely and quickly among manufacturers [2]. As a result, less successful competitors will be gradually driven out, leading to declining value-adjusted prices [3]. This theory generally holds true for industries, such as information technologies, mobile communications, and banking [4]. Therefore, it is believed that competition is efficient in addressing healthcare challenges [5].

Nevertheless, the unique characteristics of healthcare markets complicate the application of traditional economic theory in assessing the impact of competition, given the absence of conditions for perfect competition [6,7]. Firstly, healthcare services are considered as differentiated products [6,8], as preferences and disease patterns vary based on region and scale [9,10]. Consequently, patients receive different healthcare services at different hospitals, even in a market-clearing scenario. This makes the healthcare market monopolistically competitive. Secondly, information asymmetry is prevalent in healthcare, potentially leading to market inefficiencies. Suppliers often possess more knowledge about diseases, treatments, and demand compared to patients, giving them a dominant position. Intensified competition among hospitals in such markets could trigger a cycle of escalating medical services, resulting in higher costs for patients and unnecessary utilization of services due to supplier-induced demand [8,11,12,13]. The role of competition in the healthcare market remains under-investigated [14,15].

Traditionally, healthcare systems have been heavily regulated by national governments with very few exceptions, such as the US. Nevertheless, pro-competitive reforms have been introduced in many countries since the beginning of the 21st century, which include, for example, the corporatization of public providers, prospective payment schemes, pay-for-performance schemes, patient choice of provider, and the entry of private providers [15]. For example, in 2000, the Centers for Medicare and Medicaid Services (CMS) changed the reimbursement system for outpatient care at Federally Qualified Health Centers to include a prospective payment system for Medicaid and Medicare in the US [16]. In addition, see a scoping review by Turner and Wright for details on the corporatization of public providers [17].

Examples of national regulation include the following policy measures. In 2001, after the “patient choice” reform in Norway, patients were allowed to select any national health service (NHS) hospital across the whole country for non-acute treatments [15]. In 2002–2011, a series of changes in the market for NHS-funded hospital care were introduced in the UK with the intention of stimulating competition to improve quality and reduce waiting times [18]. In France, in 2004/2005, a payment reform where all hospitals are paid by fixed diagnosis-related group-based prices was introduced. This payment reform promoted benchmarking competition between public and private hospitals because hospital revenues were linked to patient volume [19]. In addition, since 2008/2009, minimum activity thresholds were used for regulating access to the cancer market [20]. In 2006, the healthcare system in the Netherlands underwent extensive reform when managed competition was introduced, “*to reduce costs, increase the quality and accessibility of healthcare and, at the same time, to maintain an equitable healthcare system*” [21] (p. 5). In Germany, public reporting was implemented in 2008 for all acute care hospitals to foster fair competition for the best quality of care [22]. In many national healthcare markets, integrated care and competition have emerged simultaneously. Examples include the introduction of an integrated delivery system [23] and managed care [24] in the US under a framework of privatization and competition and the introduction of integrated care in quasi-market or regulated markets in the UK [25,26], Sweden, and Germany [27]. These reforms provide a basis for hospital competition, especially by quality of care.

This study addresses the research question: How does hospital competition affect the quality of care? Additional questions are: Which contexts are associated with positive and negative effects of hospital competition on the quality of care? What are the differences in these effects across different time spans? In which areas, e.g., composition of services, quality, price, costs, and for which types of medical care services, is competition stronger/weaker, and why?

## 2. Defining Key Terms

This review covers the following key concepts. The first concept is ‘competition’, and, in our context, ‘hospital competition’. Competition refers to choice of medical care providers, when the choice is made by payers or consumers of medical care [28]. In these situations, care providers have an opportunity to influence the results of this choice, and the results of the choice determine the amount of financial or other material resources received by providers from payers. There are six types of choice situations in which competition among healthcare providers might simultaneously occur [28]. The first group refers to the selection of healthcare providers by the payer of healthcare in the public health financing system. It includes (1a) administrative competition when the payer is a healthcare regulatory body to which providers are administratively subordinated; (1b) competition in the internal market when payers are healthcare authorities to which providers are not administratively subordinated, and (1c) competition in a quasi-market when the payer is (are) an insurer (or several insurers). The second group of choice situations refers to (2) patients’ choice of healthcare providers in the public health financing system. The third group refers to healthcare payer selection of healthcare providers in private healthcare financing systems. These situations include (3a) competition for patients in private healthcare systems when the payer is a voluntary health insurer, and (3b) competition for patients in the medical services market when the payers are patients. Another major category of hospital competition, which considers the supply side, is cost competition [3].

Typically, researchers measure hospital competition using the following indicators: (market) concentration [29], usually measured by the Herfindahl–Hirschman index (HHI) [15,20,21,30,31], and the number of hospitals in the market [20,31,32,33]. In some studies, competition is proxied by physician density [34], doctor-to-population ratio [35], distance to other general practitioners [36], the degree of perceived competition [37], or even instrumented by, for example, the marginality of local English Parliamentary seats [38]. In their study, Wong et al. examined whether different hospital competition measures matter in empirical investigations of hospital behavior [39]. As most of the measures are highly correlated, they cautioned against using arbitrarily selected competition measures if the magnitude of the estimates is important.

Hospitals can be classified by size (small hospitals with fewer than 100 beds, medium hospitals with 100 to 499 beds, large hospitals with at least 500 beds), by duration of care provided (acute vs. long-term care hospitals), by ownership type (for-profit, not-for-profit, government-owned hospitals), by location (rural vs. urban hospitals), by university affiliation (academic/university/teaching vs. general/non-teaching hospitals), by funding source (federal, state/region, or local hospitals), etc. [40]. These characteristics affect supply-side care and hospital competitive strategies, as clarified later in this section.

The second concept is ‘quality of care’. It includes the following categories: input quality, process quality, and output quality [41]. Researchers usually assess quality of care using objective indicators including, but not limited to, the number of tertiary hospitals, the number of hospital health personnel per 1000 population, and the private share of hospital beds [42]. Objective output quality indicators include: mortality following acute myocardial infarction (AMI mortality) [43], pneumonia mortality [44], hip fracture mortality and stroke mortality [45], risk-adjusted stroke mortality [31], all-cause mortality of emergency patients and hospital readmission rates [15], emergency readmissions (for hip and knee replacement), and emergency readmissions (for coronary bypass) [18], the mean number of outpatient revisits [46], (a) the risk of in-hospital death and (b) ambulatory care sensitive condition hospitalization (for patients with hypertension) [35]. Notably, Wadhera et al. consider patient revisit numbers as an important measure of both process care quality and output care quality [47]. Similarly, Martsolf et al. argue that patients’ revisits indicate the healthcare needs that were not met during the initial visit or follow-up visit, and a high number of revisits exacerbates the resource constraints of healthcare institutions [48]. Additional objective output quality indicators include length of stay [49], pre-operative length of stay (for hip and knee replacement patients) [50], inpatient complications [24,51,52], wait times [53], and the number of violations and penalties [54]. In a few studies, subjective output quality indicators, such as patient-reported health outcomes (PROMs) (before and after the non-emergency surgery) are utilized [55,56]. Proxies of ‘process quality’ at hospital level include the likelihood of providing innovative surgical procedures [31] and aggregated indices calculated in accordance with a system of quality indicators from the ‘Dutch Healthcare Transparency Program’ [21]. In this systematic review, we focus on non-price competition and all dimensions of care quality.

Previous research identified four major factors that may moderate the relationship between hospital competition and care quality (cf. Berta et al.) [57]: the institutional settings of hospital care, i.e., market supply side [43,58,59,60], the degree of information on hospital care quality [61,62], the degree of patient freedom of choice [58,63,64,65,66,67], and hospitals’ competitive strategies [43,58,65].

## 3. Overview of the Relevant Reviews

Supplementary Table S1 summarizes the main characteristics of 12 recently published systematic reviews. These characteristics include the type of review, focus, countries covered, sample period, the number of studies reviewed, databases searched, and main results. These reviews cover evidence coming mainly from high-income countries.

Ghiasi et al. reviewed the impact of hospital competition on the strategies and outcomes of US hospitals in 1996–2016 exploring 143 relationships from 65 studies. Their results concerning statistical associations are mixed: “*almost half of them found [a] significant relationship between hospital competition and various outcome measures (35 positive and 38 negative), whereas the remaining 70 (or 49%) did not find any significant association*” [68] (p. 23).

Shen et al. focused on the potential implications to care for senior patients in their systematic review and meta-analysis, which covered the studies published between 2003 and 2013. According to their results, hospital competition was associated with a slight, but not statistically significant, increase in AMI mortality rates in Australia, the Netherlands, the UK, and the US. They conclude that, “*older patients with complex care needs may be at risk for poorer quality of care related to hospital competition*” [69] (p. 263).

Jamalabadi et al. provided a synthesis of research published between 1990 and March 2019 concerning the relationship between hospital cost/price and the quality of care in 15 OECD countries, which demonstrated no general relationship [70].

Jiang et al. conducted a systematic review and meta-analysis of studies published before September 2019 to assess the impact of hospital-market competition on unplanned readmission in Australia, South Korea, Taiwan, and the US. The pooled results of three heterogeneous studies demonstrated that it was uncertain whether hospital competition reduces readmission, while inconsistent results were found in the remaining six eligible studies [71].

Mariani et al. conducted a systematic review of studies published before January 2020 to assess the impact of hospital mergers on healthcare quality measures in Czechia, Denmark, Norway, Sweden, the UK, and the US. They documented “*inconsistent findings and few statistically significant results*” [72] (p. 191). In addition, the analyzed measures demonstrated “*an insufficient strength of evidence to achieve conclusive results*” [72] (p. 191). Nevertheless, their results identified a tendency for a decrease in the number of beds, hospital personnel, and inpatient admissions, and an increase in mortality and the readmission rate for AMI and stroke [72] (p. 191). Similarly, Mills et al. [73], Mullens et al. [74], and Stansberry et al. reviewed the impact of hospital closures on rural communities in the US. Predictably, “*as hospitals close, travel times increase cumulatively, reduce access to care, and, in turn, increase the risks associated with time-sensitive health events. [...] The loss of rural hospitals may also increase mortality and morbidity in vulnerable communities and the overall health system through interrelated effects on bystander hospitals, the availability of healthcare providers, individual and community socioeconomic status, and community well-being*” [75] (p. 13).

Reviews by Zander et al. [76], Ahmed et al. [77], Bradow et al. [78], and Yang et al. [79] are less relevant to our research question as they focus on competition among physicians, general practitioners, maternity units, and nursing homes, respectively (rather than hospital competition per se). Thus, there is a research gap resulting from a lack of empirical studies published within the last 10 years on the impact of hospital competition on quality of care in Europe.

## 4. Methods and Data

Following the PRISMA reporting guidelines [80], we employed a systematic review to identify and classify all the literature that is related to our research questions. The protocol of this systematic review was published in the Open Science Framework (OSF) with the following digital object identifier (DOI): 10.17605/OSF.IO/AW3XJ. The databases used in the study are ScienceDirect, PubMed, Wiley Online Library, and Google Scholar. The search used the following key terms and/or MeSH terms: {“competition” OR “competitiveness” OR “market pressure” OR “rivalry” OR “physician density” OR “physician supply” OR “merger” OR “closure”} AND {“care”} AND {“hospital” OR “health facility” OR “medical organization”} AND {“quality of care” OR “hospital performance”} AND {“regression” OR “correlation”}. We focused our search on articles in English, published between January 2015 and mid-April 2024.

**Figure 1:**
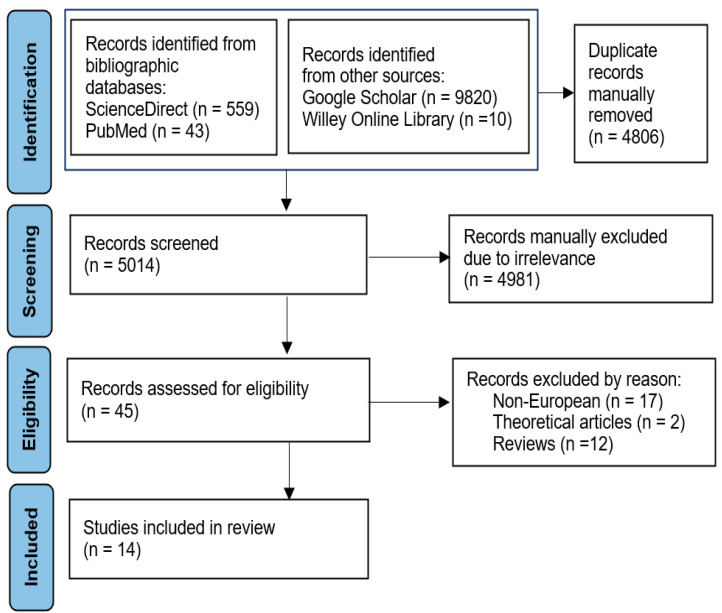
Flow diagram of selecting articles for review.

We included publications if they were peer reviewed, they included empirical data, they had extractable data related to hospital or medical care, and they could be classified as either “research article” or “original research”. We excluded publications of the following types: expert opinion, single or multiple case studies, conceptual theoretical study, editorial, commentary, letter to the editor, short communication, study protocol, and policy paper. We also excluded studies based on qualitative data, data from laboratory experiments, or data from outside of Europe. The PRISMA diagram demonstrates details of our search for eligible studies (see Figure 1).

The literature was reviewed independently by two researchers (M.J. and Y.T.) using the criteria outlined above, and queries on the suitability of individual studies were discussed with their colleagues (V.G. and E.M). Reference lists of selected publications were screened for any further potentially relevant resources. AMSTAR Checklist, a 16-question measurement tool to assess systematic reviews, was used for quality assessment of the studies. In addition, insights on search procedure from a recent study on hospital efficiency [81] guided our selection process.

## 5. Results

### 5.1. Descriptive Statistics

The initial search returned 9820, 559, 43, and 10 papers in Google Scholar, ScienceDirect, PubMed, and Wiley Online Library, respectively. The removal of duplicates yielded 5014 articles. The selected papers were screened by title and subsequently screened on their abstract or full text. In total, 14 studies were selected for review based on the criteria listed above, covering France (2), Germany (1), Italy (3), the Netherlands (2), Norway (1), Sweden (2), and the UK (3). In eight articles, HHI is used among hospital competition measures. Objective output indicators dominate among the indicators of care quality (in 11 articles), with two instances of objective process indicators, and one study with subjective output indicators. (There are instances of simultaneous use of two types of indicators). In 13 studies, parametric methods, including difference-in-differences design (5) and multilevel models (5) are employed, while one study utilizes non-parametric tests only. Table 1 summarizes the articles, which were included in the analysis.

### 5.2. The Effects of Hospital Competition on Care Quality in Europe

The first group of results is based on applying econometric models with relatively standard sets of proxy variables for hospital competition and care quality to the data from the respective counties.

#### 5.2.1. Germany, France, the Netherlands, and Italy

#1. A study by Strumann et al. considered the relationship between the examined phenomena through a lens of public reporting (PR) on healthcare quality as PR aimed to steer patients towards high-quality providers and promote fair competition based on quality [31]. The researchers focused on the impact of the initial public release of performance data in Germany in 2008. Given the intricate nature of stroke patient care, overall hospital quality was proxied by the 30-day risk-adjusted mortality rate for stroke treatment. Market competitiveness was computed using predicted market shares derived solely from exogenous factors to allow for a causal understanding of the impact of the reform. The study revealed a homogenous positive relationship between competition and care quality. The observed effect was mostly determined by not-for-profit hospitals specializing in a narrow range of services and for-profit hospitals offering a medium range of services. In addition, the results suggested that non-specialized hospitals, which play a crucial role for local emergency and acute care, might not be systematically able to increase their quality in response to the competitive pressure of PR. In contrast, the highest-quality improvement effects were attributed to medium private for-profit and highly specialized not-for-profit hospitals. This difference might be explained by the distinct necessities and opportunities of the various hospital types to enhance their quality. The different ownership types and degrees of specialization potentially determine the hospitals’ flexibility and resources (allocated to maintain their competitive position).

Despite this homogenous effect, several structural reasons potentially limit quality competition in Germany (although legally free patient choice of hospitals exists). They include: (a) limited patient mobility; (b) patient “path-dependence” (i.e., a fact that patients often choose hospitals that they have previously attended or their outpatient physicians have recommended); (c) patient choice can be exercised if they have an option among different hospitals offering the necessary medical service. In general, the findings demonstrate the significance of outcome transparency in improving hospital quality competition.

#2. Or et al. analyzed shifts in market competitiveness and treatment patterns within the realm of breast cancer surgeries done in 2005 and 2012 [20]. They focused on the adoption of technology as an indicator of procedural excellence, investigating the probability of providing immediate breast reconstruction (IBR) post mastectomy and sentinel lymph node biopsy (SLNB). Employing a competition index derived from a multinomial logit model of hospital selection, which mitigates endogeneity bias, they assessed its influence on the likelihood of administering IBR and SLNB through multilevel models that considered both observable patient and hospital attributes. The probability of undergoing these interventions is notably elevated in hospitals situated in more competitive markets. Nevertheless, hospital caseloads continue to serve as a significant metric of quality, thereby suggesting that the advantages of competition are contingent on the evaluations of caseload impact on treatment protocols. In France, the implementation of a centralization policy, with specified minimum activity thresholds, has played a role in enhancing the treatment of breast cancer during the observed period. The results revealed that markets bordering on monopolistic structure do not foster advancements or excellence in cancer therapy. However, excessively competitive markets characterized by numerous hospitals with minimal activity levels also present issues as the quality of hospital services is positively correlated with the number of patients.

#3. Goro et al. conducted a retrospective cross-sectional study from 2015 to 2019 utilizing data from a National Health Database [82]. Patients operated on for colorectal cancer in hospitals in mainland France were included. The results demonstrated that increasing hospital competition independently decreased the 30-day mortality rate after colorectal cancer surgery. Other factors significantly associated with mortality included age, gender, patient comorbidity index, malnutrition, neo-adjuvant treatment, the emergency admission, type of surgical procedures, and hospital caseload. The findings were robust when alternative competition measures were used. However, future research would benefit from controlling for surgeon and hospital quality to avoid a potential omitted variable bias.

#4. Croes et al. examined the correlation between quality assessments for three specific diagnosis groups and hospital market share [21]. The analysis focused on assessing the effect of competition on quality within a context of deregulated pricing. The investigation leveraged distinct pricing and output data pertaining to the three diagnosis groups (cataract, adenoid and tonsil, and bladder tumor) provided by Dutch hospitals between 2008 and 2011. Quality metrics associated with these diagnosis groups were also utilized. For cataract and bladder tumor, the relationship between hospital competition and quality scores was significant and robust. For adenoid and tonsils, no significant associations were found. One possible explanation is that the patient group for adenoid and tonsil is less complex: It is mainly children under 11. This type of patient has fewer additional diagnoses compared to patients with, for example, bladder tumor. Endogeneity concerns prevent price from being included as an independent variable in the quality indicator models, suggesting the absence of the relationship between price and quality scores: hospitals with higher-quality scores do not have higher prices. Summing up, the findings indicated an inverse relationship between market share and quality assessment for two out of the three diagnosis categories, suggesting that hospitals operating in competitive environments exhibited superior quality assessments.

#5. Van der Schors et al. utilized data from Cancer Registry in the Netherlands to examine the impact of hospital volume and competition on outcomes for invasive breast cancer (IBC) surgery patients between 2004 and 2014 [33]. According to the results, treatment types, patient, and tumor characteristics mainly explained variations in outcomes. Hospital volume and competition did not significantly affect surgical margins or re-excision rates after adjusting for variables. Survival was slightly higher for patients in hospitals with higher annual surgery volumes and more competitors nearby. However, the effect of hospital competition on survival did not remain consistent after adjusting the proxy. Hospital volume and regional competition play a limited role in explaining variations in IBC surgery outcomes in Dutch hospitals. The study suggested two theoretical explanations for the absence of a robust relationship between hospital competition and care quality. First, in the Netherlands, “*the role of competition among hospitals in breast cancer care is limited through the rare use of selective contracting by health purchasers*” [33] (p.11). Competition in this market segment is not strengthened by active patient choice: most breast cancer patients agree to be referred to the nearest hospital by their general practitioner. Second, due to the potential co-existence of competition and collaboration in health systems, “*the competition-effect might be mitigated by an unobserved collaboration-effect*” [33] (p.11).

#6. Lisi et al. explored the geographical extent of the impact of hospital competition on quality, utilizing data from the Lombardy region of Italy that encompasses over 207,000 patients from 2008 to 2014 [83]. The researchers introduced an economic framework that took into account not only local forms of quality competition among hospitals, but also global aspects. Global competition arises from periodic releases of hospital performance rankings. Within this framework, they deduced the hospital reaction functions and subsequently characterized the interdependence among the quality of hospitals. This might be explained by the fact that hospital managers’ decisions on care quality are also affected by hospital outcomes from outside their local market. In line with their theoretical predictions, the empirical results revealed a significant positive degree of short- and long-term dependence. In addition, these findings indicated the presence of local and global competition dynamics among hospitals. In general, while the findings were in line with other studies (e.g., Bloom et al. [38]; Cooper et al. [65]), they also suggested that healthcare systems could benefit not only from more competitive local hospital markets, but also from making their institutional environment more competitive for hospital management.

#7. A study by Guida et al. quantified the level of competition among Italian healthcare providers and assessed the potential correlation with hospital mortality rates [84]. The sample comprised individuals discharged in 2015 after CHF or AMI, as well as those discharged in 2014 and 2015 following cardiac surgery. The findings indicated that competition indicators exhibited variability across different medical conditions and were notably sensitive to the methodology employed for defining the geographical market boundaries. Notably, neither the quantity of hospitals nor HHI demonstrated significant correlations with outcomes for CHF cases. The hospital competition measures fluctuated depending on the definition of the local market, leading to varying mortality correlations for different medical conditions. The inverse correlations between competition measures risk-adjusted mortality ratios (RAMRs) suggested the possible role of case volumes on outcomes. The study documented that “*[a] hospital’s RAMR for valve surgery increased in more competitive areas at the analyses for fixed cases volume and fixed number of hospitals*” [84] (p. 601). This inverse relation was mainly explained by the possible positive effect of high volumes. Mortality and hospital volume, being proxies for care quality, might drive patient choice of cardiac surgery center and, consequently, strengthen the relationship between these indicators. Larger volumes were possible in areas with lower competition. Consequently, high-volume hospitals were more likely to have high-volume surgeons and demonstrate better outcomes. Patients tended to choose better hospitals, as they are sensitive to care quality. In contrast to valve surgery, lower RAMRs in more competitive areas of fixed size was observed. The latter findings were similar to those observed in the English NHS: competition can raise the quality of CABG surgery [60].

#8. Berta et al. explored the influence of competition on adverse health outcomes in hospitals within an environment where information on hospital quality is not publicly disclosed [57]. The study utilized data from patients admitted to hospitals in the Lombardy region of Italy, where annual risk-adjusted hospital rankings are exclusively available to hospital administrators. Consequently, patients might base their hospital selection on factors such as proximity, local network recommendations, and general practitioner referrals. Through the estimation of patient-predicted choice probabilities, a series of competition indices were constructed to evaluate their impact on a composite index encompassing mortality and readmission rates as indicators of hospital quality. The results suggested no discernible link between adverse events and hospital competition, potentially attributed to information asymmetry (i.e., a lack of publicly available information on the quality of hospitals) and the complexities associated with developing reliable health quality indicators.

Thus, the results from Germany and partly from the Netherlands suggested the existence of a positive relationship between hospital competition and care quality. The evidence from Italy was contradictory (positive only; positive and negative; and no associations), while the findings from France was inconclusive with some evidence of a positive relationship.

#### 5.2.2. Scandinavia

#9. Brekke et al. examined the effects of introducing non-price competition to hospitals within an NHS setting, through the implementation of patient choice reform in Norway in 2001 [15]. This reform enabled the application of a difference-in-differences approach due to the plausibly exogenous (geographical) variations in the pre-reform market structure. By utilizing comprehensive administrative data encompassing all hospital admissions from 1998 to 2005, the study employed models with fixed effects for hospitals and treatments/DRG, focusing solely on emergency admissions to mitigate potential patient-selection biases. The findings indicated that hospitals in more competitive regions experienced a notable decrease in AMI mortality rates (although it is sensitive to alternative competition measures), while observing no impact on stroke mortality. Additionally, exposure to competition was associated with lower all-cause mortality rates, reduced lengths of hospital stay, and increased readmissions, albeit with minor effects. Interestingly, higher DRG prices reinforced the identified effects of competition, which appeared to reduce wait times and increase admissions. Although these findings were in line with the results from the UK (see e.g., Cooper et al. [65] and Gaynor et al. [49]), interpretation should be done with caution as elective treatments were included in the analysis, while a full welfare analysis was not conducted.

#10. A study by Goude et al. examined the effect of a pro-competitive reform that was implemented in 2009, specifically targeting elective hip replacement surgeries in Stockholm, Sweden [85]. The impact of this reform on the quality of hip replacement surgeries was assessed using various patient-reported outcome measures (PROMs) related to health improvement, pain alleviation, and patient satisfaction, 1 and 6 years after the surgery. The data encompassed elective primary total hip replacements conducted from 2008 to 2012. The adoption of entropy balancing mitigates divergences in observable characteristics and to harmonizes all covariates across the treatment groups. However, no statistically significant effects of the reform on any of the targeted PROMs at the 1- and 6-year follow-ups were identified. Notably, the introduction of competition and bundled payment schemes did not yield any discernible improvements in the quality of hip replacement surgeries, proxied by the post-surgery PROMs. Within the bundled payment model (the spine surgery program), two types of outcomes with differences in the strength of financial incentives were considered that potentially explain the lack of hospital competition effects on PROMs. First, providers are responsible for covering healthcare costs related to the hip replacement surgery, including complications such as infection and revision surgery within 2 years after surgery. Second, the performance payment, which is partly based on PROMs, is only a small percentage of the bundled payment in magnitude. Even if both outcomes are observable, healthcare providers have more financial incentives to avoid negative outcomes, e.g., complications, rather than to focus on PROMs. The findings somewhat contradicted the results of Skellern et al. [56] related to the pro-competition reform in the English NHS in 2006, who document lowered care quality measured by PROMs of health gain for hip and knee replacement. This discrepancy was explained by differences in the setting (e.g., the level of competition and design of economic incentives, and sample) and methodology.

#11. Avdic investigated how the geographical proximity to an emergency hospital affected the likelihood of surviving an AMI, while considering health-related spatial sorting and data constraints related to out-of-hospital mortality in Sweden [86]. Leveraging policy-induced shifts in hospital distances due to emergency hospital closures and extensive mortality data spanning 2 decades, the results revealed a substantial decrease in the likelihood of surviving an AMI as the residential distance from a hospital increases in the year following a closure. Nonetheless, this effect diminished in subsequent years, indicating a swift patient adaptation to the altered circumstances. The latter finding might be explained by compensatory strategies adopted by prospective patients and the healthcare administration to accommodate any distance effects following hospital closures. For example, patients with poor health who experienced reduced access to emergency healthcare might decide to move closer to the new home hospital, while healthcare authorities might ex post invest more in emergency healthcare. In addition, distance changes are exogenous only just after the closure because patients, and healthcare authorities, can react and adapt to the changes over time. Finally, the temporary increase in AMI mortality caused by hospital closures might be compensated for by productivity gains from the subsequent consolidation of inpatient care at the remaining hospitals.

Thus, the evidence from Norway and Sweden based on objective quality indicators suggested the existence of a significant positive association between hospital competition and care quality. In contrast, the findings from Sweden did not reveal any effects, when subjective PROMs were used to measure care quality with respect to hip replacement surgeries, which are less dangerous compared to AMI.

#### 5.2.3. The United Kingdom

#12. Moscelli et al. examined the impact of a policy reform that relaxed constraints on patient selection of hospitals on the quality of three high-volume non-emergency medical treatments in the UK [18]. The quasi difference-in-differences strategy and integrate control functions that allow for the possibility of patient self-selection into providers, which might be correlated with unobservable morbidity factors. The sample comprised patients funded by the NHS who received treatment in NHS-affiliated hospitals. The analysis revealed that public hospitals operating in environments with heightened competition experienced a decline in quality, prolonged wait times, and reduced lengths of stay specifically in the context of hip and knee replacement surgeries. This phenomenon was attributed to regulated pricing mechanisms that resulted in larger financial losses for these procedures compared to coronary artery bypass grafts, where no discernible effects were observed. Importantly, the findings exhibited robustness across different estimation methods and competition metrics, even when accounting for the entry of private healthcare providers into the market. The results for elective care were also compatible with those for emergency care, which use a similar identification strategy, although they found that the choice reform reduced mortality for AMI [49,65] and hip fracture [45] for hospitals with more competitors. If emergency mortality was used as a reliable indicator of overall hospital quality by patients undergoing elective treatments, then patient choice could increase emergency quality and reduce elective care quality as the result of diverted effort. In addition, the reductions in mortality were likely to have generated health benefits that were larger than the health losses for patients undergoing elective treatments as measured by higher emergency readmissions for hip and knee replacement patients. The observed reductions in quality for knee and hip replacement surgeries do not mean that the 2006 choice reform is welfare reducing overall. Patients undergoing elective procedures might benefit from an opportunity to switch to previously unobtainable providers of better quality.

#13. The primary objective of the national longitudinal study conducted by Aggarwal et al. was to analyze the association between patient choice and hospital competition in relation to post-operative outcomes following prostate cancer surgery [51]. The study encompassed the entire male population who underwent prostate cancer surgery in the UK from 2008 to 2011. The study assessed the impact of a radical prostatectomy center’s placement in a competitive setting and its effectiveness in attracting patients from other healthcare facilities on three patient-centered metrics. The findings indicated that, even after adjusting for individual patient attributes, males who underwent surgery at centers in more intensely competitive environments exhibited a reduced likelihood of experiencing a 30-day emergency readmission, regardless of the nature or quantity of procedures conducted at each respective center. Furthermore, individuals receiving care at centers that attract patients from other hospitals were less likely to have a length of hospital stay more than 3 days. The findings suggested that hospital competition contributes positively to short-term post-operative outcomes following prostate cancer surgery. The association between being a successful competitor and a reduction in hospital length of stay might be explained by two main factors. First, these specialized medical centers are more likely to be higher-volume centers compared with unsuccessful rivals. Second, during the period of analysis, successful competitors adopted robot-assisted radical prostatectomy more rapidly, which was likely to determine better outcomes.

#14. Moscelli et al. analyzed the consequences of relaxing constraints on patient choice of hospitals within the NHS in 2006, particularly focusing on its influence on mortality rates [45]. The research utilized a comprehensive data set spanning from 2002 to 2010, encompassing three critical emergency conditions associated with high mortality risks: AMI, hip fracture, and stroke. Given the varying mortality risks associated with different sub-diagnoses of AMI and stroke, the analysis incorporated indicators for sub-diagnoses within the covariates. In addition, the study accounted for potential variations in the impact of covariates on mortality rates before and after the 2006 choice reform. The findings revealed that the choice reform resulted in a slight reduction of mortality risk for patients with hip fractures. However, the reform exhibited statistically insignificant negative effects on mortality rates for patients with AMI and stroke. The study highlighted heterogeneous effects across different sub-diagnoses of AMI and stroke. The decline in mortality rates for hip fracture cases was more pronounced among more deprived patients. These findings were in line with the results of the previous studies on this reform [49,65] and highlighted the nuanced nature of pro-competition policies, emphasizing the importance of considering the interplay between patient sample and institutional setting. Theoretical models offered additional explanations for the difference in the results across type of condition. They demonstrated that higher competition and patient choice could either increase or decrease care quality, depending on the characteristics of supply and demand, the hospital objectives, and the level of price regulation.

Thus, the results from the UK are heterogeneous and contradictory: in two studies, positive and no relation between competition and objective care quality indicators are documented, while the most recent article argues in favor of a negative association between the examined phenomena in certain contexts.

Summing up, eight out of the 14 reviewed studies documented significant positive associations, at least in the short term, between hospital competition and quality of care (measured through objective outcome indicators). Of the other six, one study demonstrated a negative relationship in a specific context. The findings of the remaining five studies were heterogeneous and context-dependent (two) or suggested no discernible association between the two examined phenomena (three). The results should be treated with caution as our sample is small, and the measures of competition and care quality vary among these studies.

The following contexts were associated with a positive relationship between hospital competition and quality of care: stroke in Germany, in 2006 and 2010; colorectal cancer in a hospital in mainland France between 2015 and 2019; breast cancer treatment in France between 2005 and 2012; adenoid and tonsils, bladder tumor, and cataract in the Netherlands between 2008 and 2011; ischaemic stroke, haemorrhagic stroke, hip fracture, hip replacement, and knee replacement in Lombardy (Italy) between 2008 and 2014; AMI, stroke, and all-cause mortality in Norway between 1998 and 2005; AMI in Sweden between 1990 and 2010; and prostate cancer surgery in the UK between 2008 and 2011; in an area with a fixed radius of 100–150 km, after isolated-coronary artery bypass graft procedures in Italy in 2015; and AMI, stroke, and hip fracture in the UK between 2002 and 2010. In contrast, a negative association was observed in the following contexts: hip replacement, knee replacement, and coronary artery bypass grafts in the UK between 2002 and 2011; and after AMI and valve surgery in areas identified by the variable-radius method in Italy in 2015. No statistically significant associations were found in the following contexts: invasive breast cancer in the Netherlands between 2004 and 2014; cardiac surgery, cardiology, and general medicine in Lombardy (Italy) in 2012; hip replacements in Sweden between 2008 and 2012; chronic heart failure in Italy in 2015; and AMI and stroke in the UK between 2002 and 2010.

## 6. Discussion

To begin with, the relatively low number of eligible articles does not allow any statistical assessments regarding the examined effect of hospital competition on care quality to be made. A formal meta-analysis is hindered by the overrepresentation of studies from Italy and the UK and by the heterogeneity in the methods used in the studies. Second, identified contexts, which are associated with positive, negative, heterogeneous, or no associations between hospital competition and care quality, do not reveal the reasons behind these relationships. Third, our results cannot be directly compared with the results of previous studies as they differ in several major characteristics.

Brekke et al. report that there is “*a lack of rigorous empirical evidence on the causal effects of exposing health care providers to competition, especially from outside the US and the UK, and the existing evidence on the impact of competition is generally mixed*” [15] (p. 2). This review provides new evidence from European countries documenting instances of conflicting results in within- and between-country dimensions.

In general, the findings of earlier studies from Germany [34,87], the Netherlands [88,89,90], and Russian-speaking countries [91] are in line with our results. For example, Sundmacher and Busse document that, “*better accessibility or quality of care might have linked increased physician density with improved health outcomes*” [87] (p. 60). Jürges and Pohl found weak and insignificant effects of physician density on care quality measured as the degree of adherence to medical guidelines [34]. Bijlsma, Koning, and Shestalova demonstrated that Dutch “*hospitals facing more competition organize diagnostic processes more efficiently*” [88] (p. 121). Ikkersheim and Koolman provided evidence that, “*hospitals that faced more competition from geographically close competitors showed a more pronounced improvement [of Consumer Quality Index scores]*” [89] (p. 1). Heijink, Mosca, and Westert found “*limited between-hospital variation in quality and there was no clear-cut relation between prices and quality*” [90] (p. 142).

Similarly to the results of our review, evidence of previous studies from the UK is mixed. Cookson, Laudicella, and Donni found a negative association between market competition and elective admissions in deprived areas: “*The effect of pro-competition reform was to reduce this negative association slightly, suggesting that competition did not undermine equity*” [92] (p. 410). Cooper et al. documented that “*after the reforms were implemented, mortality fell (i.e., quality improved) for patients living in more competitive markets*” [65] (p. F228). Propper, Burgess, and Green report that, “*the relationship between competition and quality of care appears to be negative. However, the estimated impact of competition is small*” [59] (p. 1247).

The results of empirical studies from Africa and Asia, which utilize objective output quality indicators, are fragmentary and inconclusive, suggesting a positive association for hypertension patients in Ghana [35] and for participants with haemorrhoids in South Korea [93,94], a negative association for pneumonia patients in China [95], a negligible negative effect for stroke patients in Taiwan [96], and an insignificant correlation with AMI in-hospital mortality in China [95]. A rapidly rising number of large Chinese hospitals with a capacity of more than 2000 beds [97] might explain recently documented heterogeneous effects of hospital competition on inpatient quality in this country [98].

In general, according to theoretical predictions, the following factors might explain the variability in the relationship between hospital competition and care quality: the institutional settings of hospital care [43,58,59,60], the degree of information on hospital care quality [61,62], the degree of patient freedom of choice [58,63,64,65,66,67], and hospitals’ competitive strategies [43,58,65].

In addition, our results suggest that there are countries, such as France, Germany, and Norway, where only one type of relationship (positive) between hospital competition and care quality is observed regardless of the context. Therefore, it might be hypothesized that healthcare systems in which patients are free to choose their provider (France, Germany) and where hospitals are very heterogeneous in terms of type and the range of services (France, Germany) are likely to experience positive effects of hospital competition on quality of care. The same might be true for systems with universal public health insurance with 95% to 100% of the hospital expenditure covered by taxes (France, Norway).

Finally, theoretical predictions suggest the following. “*[…] Quality levels seem to be highly dependent on the market structures that are in place. Hospitals providing comparable health services may vary in the level of price-quality provided. [...] In price-regulated markets, quality is the only dimension on which one can compete. In this setting, quality levels depend on whether price-cost margins are positive or negative (Gaynor and Town, 2011). In non-price-regulated markets, where providers are able to determine price and quality level, quality levels depend on elasticities of demand for price and quality. If quality information is not transparent, competition will focus on the price dimension (Propper et al., 2004)*” [21] (p. 7). In countries where hospitals and insurers bargain with each other (e.g., The Netherlands), the bargaining framework introduced by Gaynor and Town is the most useful theoretical model for analyzing the relation between competition and quality [7].

## 7. Conclusions

More than half of the analyzed studies document significant positive association, at least in the short term, between hospital competition and the quality of care. The findings of the remaining six studies are heterogeneous and context-dependent or suggest no identifiable association between the two examined phenomena. Recall that the observed contradictions with the results of previous studies are explained by differences in institutional settings (for example, the level of competition and/or the design of economic incentives), samples, and methods.

Policymakers should understand that regulatory impact assessment (RIA) is necessary before a pro-competitive policy intervention to get a clear vision of its benefits and drawbacks for all stakeholders. At country level, the requirements to conduct RIA varies. For instance, in the UK, impact assessments are compulsory for all new policy initiatives and interventions, both regulatory and non-regulatory [99]. Yet, there is a room for improvement in RIA in Europe because only Austria, Estonia, France, Italy, Poland, and the UK have strong ex ante requirements in place to ensure that achievement of the regulation’s goals are assessed [100]. Furthermore, evidence-based policymaking can be useful for performing a periodic assessment of outcomes and processes and for adopting the appropriate corrective measures, if necessary [72]. In addition, enhancing service transparency can stimulate improvements in care quality. In Germany, for example, several websites, such as Weisse-Liste.de, report structural information on inpatient numbers, diagnoses, procedures, and risk-adjusted quality indicators in a publicly accessible and user-friendly manner, imposing potential pressure on competing hospitals [31]. Similar websites exist in almost every European country, although the extent of available information on hospital quality varies significantly among countries. To avoid cost-cutting decisions by hospital management in response to excessive competition, a balance between pro-competitive policy initiatives and regulation is essential for optimal healthcare outcomes.

We see the following directions for future research to overcome the limitations of our study. First, although quality indicators published for emergency treatments are considered as good clinical quality markers [15] (p. 10), similar research with the simultaneous use of alternative quality indicators which cover multiple dimensions would shed light on the relationship between hospital competition and quality of care. Second, inconclusive findings might be “*due to the use of different methodologies, hospital competition measures, and hospital quality measures*” [101] (p. 263). In this respect, testing alternative measures, such as the number of competitors per distance or per region (for local and global competition, respectively), geographical or time distance from the home of a patient and a hospital, along with traditional HHI, would be beneficial in shedding more light on the examined relationship as potentially more robust results might be obtained. Combining these two directions would be the most insightful, methodologically and practically. Third, further research on the impact of competition in different types of specialized care is necessary to identify the nuances of competition effects in this sector. Fourth, a meta-analysis could provide robust quantitative evidence when more data become available. Finally, relevant evidence from countries of the Global South [102] would uncover the specific effects of competition on quality of care in this region.

## Figures and Tables

**Table 1:** Summary of empirical research on the effects of competition on quality of care among hospitals.

№	Country	Year, Authors, Journal, Link	Sample	Methods	‘Competition’ Proxy	‘Quality of Care’ Proxy	Quality Aspect	Main Results	Institutional Setting
1	Germany	2022, Strumann et al., *Eur J Health Econ* [31]	Hospital-level panel data from 947 hospitals (n = 1894); 2006–2010	Difference-in-differences	(1) Actual HHI, (2) predicted HHI, (3) the number of hospitals in the relevant market	The 30-day risk-adjusted mortality rate for stroke treatment	Objective output	“A homogenous positive effect of competition on quality of care is found. This effect is mainly driven by the response of non-profit hospitals that have a narrow range of services and private for-profit hospitals with a medium range of services”.	German hospitals provide primarily inpatient treatment and services. These hospitals are heterogeneous in terms of type and the range of services; they have a uniform DRG-based payment system. Patients are free to choose their provider.
2	France	2022, Or et al., *Int J Health Policy Manag* [20]	804 hospitals, patient-level data from 2 years: 2005 (n = 54,904) and 2012 (n = 62,250)	Multinomial logit model and [means of] multilevel models	(1) HHI based on actual hospital volumes for breast cancer surgery and volumes of any cancer surgery and (2) the hospital count within the market area as an exogenous measure of competition	The likelihood of providing innovative surgical procedures as a proxy of ‘process quality’ at the hospital level	Objective process	“The likelihood of receiving these procedures is significantly higher in hospitals located in more competitive markets. Yet, hospital volume remains a significant indicator of quality, therefore benefits of competition appear to be sensitive to the estimates of the impact of volume on care process. In France, the centralisation policy, with minimum activity thresholds, have contributed to improving breast cancer treatment between 2005 and 2012”.	France has a universal public health insurance system, with more than 95% of the hospital expenditure covered by public funding. Hospital care is provided by a large variety of public, private non-profit, and private for-profit entities. Patients can freely choose between public and private providers without a referral. Private hospitals have contracts with the public health insurance fund and are reimbursed based on regulated prices as public hospitals [20].
3	France	2024, *Goro* et al., *PloS ONE* [82]	Patients operated on for colorectal cancer in a hospital in mainland France from 726 hospitals (n = 152,235), 2015–2019	A retrospective cross-sectional study; a mixed-effect model	The number of competitors per distance	The 30-day mortality rate after colorectal cancer surgery	Objective output	“Mortality at 30 days was 3.6% and we found that the mortality decreases with increasing of the hospital activity. Using the number of competitors per distance method, our study showed that a “highly competitive” and “moderately competitive” markets decreased mortality by 31% [OR: 0.69 (0.59, 0.80); *p* < 0.001] and by 12% respectively [OR: 0.88 (0.79, 0.99); *p* < 0.03], compared to the “non-competitive” market. High hospital volume (100 > per year) was also associated to lower mortality rate [OR: 0.74 (0.63, 0.86); *p* < 0.001]. Conclusions: The results of our studies show that increasing hospital competition independently decreases the 30-day mortality rate after colorectal cancer surgery. Hospital caseload, patients’ characteristics and age also impact the post-operative mortality”.	
4	the Netherlands	2018, Croes et al., *Eur J Health Econ* [21]	Price and production data relating to three diagnosis groups from 87 Dutch hospitals (n = 474,410 patients) in the 2008–2011	Panel regression models	HHI of the insurers that a hospital faces	The quality indicators from the ‘Dutch Healthcare Transparency Program’: process and structural indicators (with a ratio scale) rather than outcome indicators	Objective process	“We reveal a negative relationship between market share and quality score for two of the three diagnosis groups studied, meaning that hospitals in competitive markets have better quality scores than those in concentrated markets. We therefore conclude that more competition is associated with higher quality scores”.	The healthcare system in the Netherlands represents a setting with managed competition and compulsory healthcare coverage. Data on hospitals’ quality of care have been accessible to the public since 2008.
5	the Netherlands	2022, van der Schors et al., *BMJ Open* [33]	136,958 patients who underwent surgery for invasive breast cancer; 2004–2014	Multilevel Cox survival regression models with hospital and year of surgery random effects	Number of proximate hospitals within a fixed radius	Surgical margins, 90 days re-excision, overall survival	Objective output	“[…] treatment types as well as patient and tumour characteristics explain most of the variation in all outcomes. After adjusting for confounding variables and intrahospital correlation in multivariate logistic regressions, hospital volume and competition from neighbouring hospitals did not show significant associations with surgical margins and re-excision rates. For patients who underwent surgery in hospitals annually performing 250 surgeries or more, multilevel Cox proportional hazard models show that survival was somewhat higher (HR 0.94). Survival in hospitals with four or more (potential) competitors within 30 km was slightly higher (HR 0.97). However, this effect did not hold after changing this proxy for hospital competition”.	The Dutch market-based healthcare system might face a trade-off between hospital volume and competition in the oncological care context, where centralization and collaboration aim to enhance patient outcomes.
6	Italy	2021, Lisi et al., *Reg Sci Urb Econ* [83]	Administrative data on all patients admitted to any hospitals in Lombardy, 120,000–244,060 observations; 2008–2014	A Conditional Auto-regressive specification (CAR) model and a Spatial Auto-regressive (SAR) model	The number of rivals within the block/within the catchment group (local competition); Between-block spatial weights (global competition)	30-day mortality rate for AMI; 30-day mortality rate for ischaemic stroke; 30-day mortality rate for haemorrhagic stroke; 30-day mortality rate for hip fracture; 1-year readmission rate following hip replacement; 1-year readmission rate following knee replacement	Objective output	“Consistently with our micro-founded framework, our results show a significant positive degree of short- and long-range dependence, suggesting the existence of forms of local and global competition amongst hospitals with relevant implications for health care policy”. “[The] results point at the presence of small but significant hospital interdependence within catchment areas. On the other hand, we observe a marked heterogeneity among local markets and quality indicators, with the majority of areas indicating that hospital qualities are strategic complements, but also a few that are strategic substitutes. Conversely, our results on global interdependence are consistent among quality indicators, indicating that in our context hospital qualities tend to be strategic complements also outside catchment areas”.	The Italian NHS is highly decentralized. Financial resources are allocated to regions which are responsible for the organization and management of their regional healthcare [83].
7	Italy	2019, Guida et al., *Int J Qual Health Care* [84]	Patients discharged during 2015 for CHF or AMI, and between 2014 and 2015 for cardiac surgery (respectively, from 662, 395 and 91 hospitals)	Cross-sectional study; Spearman correlation tests	HHI; the number of competitors	Mortality for congestive heart failure (CHF), AMI, isolated-coronary artery bypass graft (CABG), or valve surgery	Objective output	“Indicators of competitions varied by condition and were sensitive to method used for the area definition. Hospital mortality after AMI and valve surgery increased with competition in areas identified by the variable-radius method (higher rates for a greater number of hospitals or lower HHIs). In area with fixed radius of 100–150 km, competition reduced mortality after CABG procedures (lower rates for a greater number of hospitals or smaller HHIs). Neither the number of hospitals nor HHI correlated with outcomes in CHF”.	
8	Italy	2016, Berta et al., *J Royal Stat Soc* [57]	Patients who were admitted in 2012 to the cardiac surgery (n = 172,280), cardiology (n = 4,601,876), and general medicine (n = 12,700,700) wards in any public or private hospital in the Lombardy region that was financed by regional public funds	Multilevel models	HHI; Time distance from residence of patient *i* and hospital *j* (in minutes)	Hospital *j* 30-day mortality rate in ward *w*; Hospital *j* 30-day after discharge readmission rate in ward *w*; Hospital *j* composite index of adverse health outcomes (i.e., 30-day mortality or readmission) in ward *w*, Percentile rank of ward w in hospital *j* in the league table of the Lombardy quality evaluation programme	Objective output	“Our results show that no association exists between such adverse events and hospital competition. Our finding may be the result of asymmetric information, as well as the difficulty of building good quality health indicators”.	An environment where information on hospital quality is not publicly disclosed.
9	Norway	2021, Brekke et al., *J Health Econ* [15]	Administrative data, covering the universe of hospital admissions in 64 NHS hospitals (n = 382,176 emergency patients); 1998–2005	Difference-in-differences	Predicted HHI, computed using pre-reform market (patient) shares	AMI, stroke, or all-cause mortality	Objective output	“…hospitals in more competitive areas have a sharper reduction in AMI mortality but no effect on stroke mortality. […] competition reduces all-cause mortality, shortens length of stay, but increases readmissions, though the effects are small in magnitude. In years with high (DRG) prices, the negative effect on readmissions almost vanishes. Finally, exposure to competition tends to reduce waiting times and increase admissions, but the effects must be interpreted with care as the outcomes include elective treatments.	In 2001, non-price competition for hospitals within an NHS setting was introduced through the implementation of patient choice reform in Norway.
10	Sweden	2021, Goude et al., *BMC Health Serv Res* [85]	Patient-level data on elective primary total hip replacements carried in 2008–2012, pre-reform (2008) and post-reform (2009–2012), (patient demography, the surgery and PROMs at baseline and at 1 year and 6 years’ follow-up); n = 36,627	Difference-in-differences (in conjunction with entropy balancing)	(Quasi-experimental research design)	Patient-reported outcome measures (PROMs) of health gain (as indicated by the EQ-5D index and a visual analogue scale (VAS)), pain reduction (VAS), and patient satisfaction (VAS) 1 year and 6 years after the hip replacement surgery	Subjective output	“The entropy balancing was successful in creating balance in all covariates between treatment groups. No significant effects of the reform were found on any of the included PROMs at one- and six-years follow-up. The sensitivity analyses showed that the results were robust. Conclusions. Competition and bundled payment had no effects on the quality of hip replacement surgery as captured by post-surgery PROMs of health gain, pain reduction and patient satisfaction”.	In 2009, a pro-competitive reform was implemented, specifically targeting elective hip replacement surgeries in Stockholm. This reform centered around granting patients the autonomy to choose their healthcare provider, facilitating the establishment of new providers, and implementing a bundled payment model.
11	Sweden	2016, Avdic, *J Health Econ* [86]	Swedish residents (n = 817,000) who were the subject of an AMI; 1990–2010	Regression analysis (OLS)	The geographical distance in kilometers from AMI patient *i*’s home to his or her home hospital	The probability of surviving an AMI	Objective output	“Exploiting policy-induced variation in hospital distance derived from emergency hospital closures and detailed Swedish mortality data over two decades, results show a drastically decreasing probability of surviving an AMI as residential distance from a hospital increases one year after a closure occurred. The effect disappears in subsequent years, however, suggesting that involved agents quickly adapted to the new environment”.	
12	The UK	2021, Moscelli et al., *RAND J Econ* [18]	Non-emergency NHS-funded patients treated in financial years 2002/3 to 2010/11 in NHS hospital sites only. N = 414,433 (hip replacement); n = 463,953 (knee replacement); n = 114,291 (coronary artery bypass grafts). Pre-choice period: financial years 2002/3 to 2005/6. Post-choice period: financial years 2006/7 to 2010/11	Difference-in-differences	HHI	Quality for elective hip and knee replacement and CABG by whether the patient had an emergency admission within 28 days of discharge after their initial elective procedure	Objective output	“Public hospitals facing more rivals reduced quality, increased waiting times, and reduced length of stay for hip and knee replacements. This is likely due to regulated prices implying larger losses on these treatments compared to coronary artery bypass grafts, where no effects were found. Our findings are robust to estimation methods and competition measures, allowing for private providers’ entry”.	In the UK, a policy reform was introduced that relaxed constraints on patient selection of hospitals on the quality of three high-volume non-emergency medical treatments.
13	The UK	2019, Aggarwal et al., *Cancer* [51]	Patients underwent prostate cancer surgery (n = 12,925); 2008–2011	Multilevel logistic regression	The number of centers (hospitals) within a threshold distance	Postoperative length of hospital stay > 3 days, 30-day emergency readmissions, and 2-year urinary complications	Objective output	“With adjustment for patient characteristics, men who underwent surgery in centers located in a stronger competitive environment were less likely to have a 30-day emergency readmission, irrespective of the type or volume of procedures performed at each center (odds ratio, 0.46; 95% confidence interval, 0.36–0.60; *p* = 0.005). Men who received treatment at centers that were successful competitors were less likely to have a length of hospital stay > 3 days (odds ratio, 0.49; 95% confidence interval, 0.25–0.94; *p* = 0.02). Conclusions. The current results suggest for the first time that hospital **competition improves short-term outcomes** after prostate cancer surgery. Further evaluation of the potential role of patient choice and hospital competition is required to inform health service design in contrast to the role of top-down–driven approaches, which have focused on centralization of services”.	A competitive setting determined by the number of neighboring centers within a specified distance.
14	The UK	2018, Moscelli et al., *Soc Sci Med* [45]	Patient-level data: AMI (n = 288,279 patients), hip fracture (n = 91,005 patients), stroke (n = 214,103 patients); 2002–2010	A difference-in-differences with a continuous treatment variable (market structure)	HHI	AMI, hip fracture, and stroke risk adjusted mortality	Objective output	“We find that the choice reform reduced mortality risk for hip fracture patients by 0.62% (95% CI: 1.22%, 0.01%), compared with the 2002/3–2010/11 mean of 3.5%, but had statistically insignificant negative effects for AMI and stroke. The reform also had heterogeneous effects across AMI and stroke sub-diagnoses, reducing mortality for 3% of AMI patients and 21% of stroke patients. The reduction in hip fracture mortality was greater for more deprived patients. Policies to increase competition and give patients greater choice are likely to have heterogeneous effects depending on details of patient case mix and market conditions”.	In 2006, patient choice reform was implemented within the NHS.

Note: AMI—acute myocardial infarction; CHF—congestive heart failure; CABG—isolated-coronary artery bypass graft; DRG—diagnosis related group; HHI—Herfindahl–Hirschman index.

## Data Availability

The search results are available at the following hyperlinks: Google Scholar, ScienceDirect, PubMed, and Wiley Online Library.

## Supplementary Materials

The following supporting information can be downloaded at: https://www.mdpi.com/article/10.3390/healthcare12222218/s1, Table S1: Summary of reviews on the effects of competition among healthcare facilities on quality of care.
