# Supplementary material for: The Impact of Hospital Competition on the Quality of Care in Europe: A Systematic Review
Source: Healthcare (Basel). 2024 Nov 6;12(22):2218. doi: 10.3390/healthcare12222218 (PMC11593865; doi:10.3390/healthcare12222218)
Supplement: Supplementary file 1 [file healthcare-12-02218-s001.zip › healthcare-3147674-supplementary.pdf]

Supplementary Table S1. Summary of reviews on the effects of competition among healthcare facilities on quality of care.

**№**	**Year, authors journal**	**Type of review**	**Focus**	**Countries covered**	**Sample period**	**Sample size**	**Databases searched**	**Main results**
1	2023, Mullens *et al.*, *J Rural Health* [74]	Scoping review	The impact of (rural) hospital closures on rural communities	US	until Dec. 28, 2022	20	PubMed, Embase, CINAHL, Scopus	"Key domains of adverse impacts related to rural hospital closure included emergency medical service transport, local economies, availability and utilization of emergency care and hospital services, availability of outpatient services, changes in quality of care, and workforce and community members. However, significant heterogeneity existed within these findings."
2	2023, Stansberry *et al.*, *Nursing Forum* [75]	Integrative review	The effect of US rural hospital closures on care quality and health status of vulnerable rural populations	US	2010 – 2019	26	CINAHL, PubMed, Scopus	"As hospitals close, travel times increase cumulatively, reduce access to care, and, in turn, increase the risks associated with time-sensitive health events. Ultimately, the loss of rural hospitals may also increase mortality and morbidity in vulnerable communities and the overall health system through interrelated effects on bystander hospitals, the availability of healthcare providers, individual and community socioeconomic status, and community well-being."
3	2022, Mills *et al.*, *J Rural Health* [73]	Systematic review	The impact of (rural) hospital closures on rural communities	US	Jan. 2005 – Dec. 2021	21	EMBASE, CINAHL, PubMed, EconLit, Business Source Complete	"Eighty-nine percent of the studies that examined economic impacts found unfavorable results, including decreased income, population, and community economic growth, and increased poverty. Between 11 and 15.7 additional minutes were required to transport patients to the nearest emergency facility after closures. A lack of consistency in measures and

№	Year, authors journal	Type of review	Focus	Countries covered	Sample period	Sample size	Databases searched	Main results
								definition of rurality challenges comparability across studies.”
4	2022, Yang *et al.*, *Gerontologist* [79]	Scoping review	Competition in the nursing home sector, and how it affects prices and quality of care	Australia, Canada, Denmark, Japan, South Korea, Sweden, Taiwan, UK, US	1988-2020	50	ScienceDirect, PubMed, Web of Science, EconLit, and Scopus	They found conflicting evidence on the relationship between market pressure and quality, i.e., “some studies find greater competition leading to higher quality, others find the opposite”. Specifically, according to their results, most eligible studies found that “greater competition tends to result in lower prices, although the effect is small”.
5	2022, Mariani *et al.*, *Eur J Public Health* [72]	Systematic review	The impact of hospital mergers on healthcare quality measures	US, Norway, Denmark, Sweden, UK, Czech Republic	until January 2020	16	MedLine, Scopus, Web of Science	“The 16 articles, included in the narrative synthesis, reported inconsistent findings and few statistically significant results. All indicators analyzed showed an insufficient strength of evidence to achieve conclusive results. However, a tendency in the decrease of the number of beds, hospital staff and inpatient admissions and an increase in both mortality and readmission rate for acute myocardial infarction and stroke emerged in our analysis.”
6	2021, Bradow *et al.*, *Midwifery* [78]	Integrative review	The impacts of rural and remote maternity unit closures in Australia	Australia	2010 – 2020	7	CINAHL, PubMed/ MedLine, Informit, ProQuest Central, Scopus,	“Women explicitly stated that access to local maternity services would negate many of their concerns.”

№	Year, authors journal	Type of review	Focus	Countries covered	Sample period	Sample size	Databases searched	Main results
							Web of Science, Google Scholar	
7	2021, Jiang *et al.*, *Risk Manag Healthc Policy* [71]	Systematic review and meta-analysis	The impact of hospital-market competition on unplanned readmissions	Australia, South Korea, Taiwan, US	until Sept. 17, 2019	3	PubMed, EmBase, Wiley Online Library, Web of Science, Scopus, JSTOR	Pooled results of three heterogeneous studies demonstrated that it was uncertain whether or not hospital competition reduces readmission, while inconsistent results were found in the remaining six eligible studies.
8	2021, Ahmed *et al.*, *BMJ Open Qual* [77]	Systematic review	The impact of current incentive schemes for general practitioners on quality of care in the UK and the effectiveness of these schemes	UK	2009 – 2019	35	Cochrane, PubMed, National Institute for Health and Care Excellence Evidence, Health Management Information Consortium, Embase, Health Management	"The majority of the literature focused on QOF. Its positive effects included reduced mortality rates, better data recording and improved sociodemographic inequalities. However, limitations involved decreased quality of care in non-incentivised activities, poor patient experiences due to tick-box exercises and increased pressure to meet non-specific targets. Findings surrounding competition were mixed, with limited evidence found on the use of non-financial incentives in primary care."
9	2020, Jamalabadi *et al.*,	Systematic review	The relationship between hospital cost/price and	Canada, Denmark, Finland, France,	Jan. 1990 – March 2019	47	PubMed/ MedLine, Scopus, EconLit	No general relationship was found.

№	Year, authors journal	Type of review	Focus	Countries covered	Sample period	Sample size	Databases searched	Main results
	Appl Health Econ Health Policy [70]		the quality of care	Germany, Japan, Spain, Finland, Hungary, Italy, Norway, South Korea, Sweden, UK, US			(ProQuest), ScienceDirect	
10	2019, Zander *et al.*, *J Health Care Organization, Provision, Financing* [76]	Scoping review	How patients choose their physician for continuous outpatient care and why they are willing to bypass the nearest physician	Netherlands, Germany, China, Denmark, Ghana, India, Norway, Scotland, UK, US	within the last ten years, no later than July 2018	17	PubMed/ Medline, ScienceDirect, Ovid Medline	"First, we extracted methods for assessing distance traveled and bypassing. Second, wei identified determinants that directly influence the traveled distance and transferred all into a conceptual framework. The center of this framework is the individual "willingness-to-go", which reflects the willingness of patients to accept additional distances. Our findings can support studies on patient mobility and physician choice, which are essential for examining both the distribution and use of medical services, as well as for adequate need related planning."
11	2019, Shen *et al.*, *Archives of Gerontology and Geriatrics* [69]	Systematic review and meta-analysis	The effect of hospital competition on quality of senior care	Australia, Netherlands, UK, US	2003 – 2013	11	PubMed, Google	Hospital competition slightly increased mortality rates of AMI, but not statistically significant. They conclude that "older patients with complex care needs may be at risk for poorer quality of care related to hospital competition".

**№**	**Year, authors journal**	**Type of review**	**Focus**	**Countries covered**	**Sample period**	**Sample size**	**Databases searched**	**Main results**
12	2017, Ghiasi *et al.*, *J Health Care Finance* [68]	Systematic review	The impact of hospital competition on strategies and outcomes of US hospitals	US	Feb. 1996 – March 2016	65	Embase, PubMed, Scopus, Google Scholar	"...concerning statistical relationships, out of 143 explored relationships, almost half of them found a significant relationship between hospital competition and various outcome measures (35 positive and 38 negative), whereas the remaining 70 (or 49%) did not find any significant association."

2
